# The Correlation Between Cognitive Flexibility and Learning Strategies Adopted by Medical Students

**DOI:** 10.7759/cureus.47560

**Published:** 2023-10-24

**Authors:** Nazia Begum, Karthika Priyadharshini Udayakumar, Kavitha Ukkirapandian

**Affiliations:** 1 Department of Physiology, Sri Venkateshwaraa Medical College Hospital & Research Center, Pondicherry, IND; 2 Department of Physiology, Jawaharlal Institute of Postgraduate Medical Education & Research, Pondicherry, IND; 3 Department of Physiology, Meenakshi Medical College Hospital & Research Institute, Chennai, IND

**Keywords:** self regulated learning, trail making test, stroop test, learning strategy, cognitive flexibility

## Abstract

Background and objective

The term cognitive flexibility refers to the ability of the students to adapt to a challenging environment. This quality has been found to enhance creativity and skills for innovation among medical students who are expected to face a taxing environment in clinical settings. Medical students should be competent enough to address the problems on their own and work with autonomy. The practice of self-regulated learning (SRL) can be associated with cognitive flexibility. Hence, this study aimed to determine the correlation between learning strategies and cognitive flexibility. Our primary objective was to correlate the different learning strategies adopted and cognitive flexibility among medical students.

Material and methods

This descriptive cross-sectional study was conducted at Sri Venkateshwaraa Medical College Hospital and Research Center, Ariyur, Pondicherry after obtaining institutional ethical committee approval. Students from the second year to the final year of the MBBS course who volunteered to participate in the study were selected based on inclusion and exclusion criteria. The Motivated Strategy for Learning Questionnaire (MSLQ), consisting of 50 items in Part B, was employed to assess SRL. Cognitive flexibility was measured using the Stroop Color and Word Test (SCWT) and Trail Making Test (TMT) Part A and Part B.

Results

The study included a total of 220 medical students. The mean age of the participants was 21.76 ± 1.77 years, and they had a healthy mean BMI of 21.06 ± 1.25 kg/m^2^. There was no significant difference in terms of gender in the tested variables. Responses in Card “C” and Card “CW” of the Stroop test showed a significant positive correlation (p<0.001) with subscales of SRL strategies. In the TMT, the latency of Trail A showed a significant negative correlation (p<0.001) with all the subscale scores of the SRL strategies, and the latency of Trail B showed a negative correlation with rehearsal (p=0.03), organization (p=0.03), and effort regulation strategies (p=0.01) of SRL.

Conclusion

Implementing SRL techniques can ultimately help medical students to act more wisely and judiciously. Hence, we propose that cognitive flexibility among medical students can be enhanced by adopting SRL strategies.

## Introduction

The concept of cognitive flexibility is defined as the ability of the brain to shift from one idea to another and adapt to changing environmental stimuli [1]. An individual's level of cognitive flexibility determines how quickly they can shift from one dimension of thinking to another dimension, which indicates the executive function of the brain. Greater cognitive flexibility is required in the medical profession to deal with the different clinical scenarios in all kinds of settings. Healthcare crises such as the coronavirus disease 2019 (COVID-19) have highlighted the importance of cognitive flexibility not only among medical professionals but also the general public.

Self-regulated learning (SRL) is defined as an active process where learners themselves set goals for their learning. Setting goals and putting in efforts to achieve them, as well as self-monitoring and time management are all examples of self-regulatory behaviors [2]. One study found that many college students lacked autonomy and commitment [3]. Practicing physicians have a social responsibility to stay competent in all crises. Unfortunately, most medical schools do not ensure that medical students are equipped with SRL abilities that are probably necessary for lifetime learning.

Even though a few studies have been conducted on learning strategies, there is a paucity of data on the correlation between cognitive flexibility and SRL strategies adopted by students; hence, this study was carried out to emphasize the importance of SRL in the medical profession. In this study, our objectives were as follows: (i) to assess the SRL strategies by using Part B of the Motivated Strategy for Learning Questionnaire (MSLQ) among medical students ; (ii) to assess the cognitive flexibility using the Stroop Color and Word Test (SCWT) and Trail Making Test (TMT) in medical students; and (iii) to determine the correlation between SRL strategies and cognitive flexibility among undergraduate medical students

## Materials and methods

This descriptive cross-sectional study was conducted at Sri Venkateshwaraa Medical College Hospital and Research Center, Ariyur Pondicherry after obtaining approval from Sri Venkateshwaraa Medical College Hospital and Research Center Scientific Research and Ethical Committee (SVMC/SRC/2022/23/CTR 734) (50/SVMCH/IEC-Cert-Aug 22). The self-selected non-probability sampling method was employed in the study. The sample size was calculated using the Epi info, Version 7.2.3.1, with a confidence interval of 95% and an expected 5% margin of error; 50% of the students were expected to submit the form, from the total population of all the currently enrolled MBBS students (excluding first year and CRRI) of 470. With an anticipated 10% failure rate with regard to participating in subsequent tests, the total sample size calculated was 242. However, only 220 students ultimately participated in all the tests on cognitive flexibility fully, and their data were analyzed.

All undergraduate medical students of both genders (114 male and 106 female) enrolled in phases II and III (part 1 and part 2) of the MBBS course and aged 18 to 25 years with a normal BMI (18-24.99 kg/m^2^) and willing to participate in the study were included. Students with acute medical illnesses that could directly affect cognitive performance were excluded from the study. Written informed consent was obtained from all the participants volunteering for the study.

Assessment of SRL

The learning strategies of study subjects were measured using 50 items in Part B of MSLQ [4]. This tool has the following nine subscales: rehearsal, elaboration, organization, critical thinking, metacognition, time and study environment, effort regulation, peer learning, and help-seeking. A 7-point Likert scale was used to score each question. MSLQ is available in the public domain and hence permission is not required to use it for research purposes.

All nine subscales were scored separately by using the following formula:

Subscale score = total score of all items in subscale/number of items in subscale.

A score of more than 3 in each subscale indicates better SRL. If the subscale score is less than 3 in more than six of the nine subscales, it means that the student needs attention with regard to SRL.

Quantification of cognitive flexibility

Cognitive flexibility was tested using the SCWT and TMT as explained below. All the tests were performed in the morning from 9:00 to 10:00 a.m. in the Department of Physiology. Proper instructions were given to the students, and they were made to fill out the questionnaire independently.

The Stroop Color and Word Test (SCWT)

The SCWT consists of three cards: 1. Card W (black and white words used as stimuli); 2. Card C (color squares used as stimuli); and 3. Card CW (both color and words used as stimuli). Each card includes a table with 10 rows and 10 columns, totaling 100 stimuli and the written words signify color names (red, blue, green, and yellow).

To measure cognitive flexibility, each subject was given Card W, Card C, and Card CW in that order. They were asked to read out the words (color names) aloud from the first card (Card W) and identify the colors from the second card (Card C). The third card (Card CW) displays words referring to the names of the colors, printed in conflicting ink colors, and it tests the ability of the participant to perform a less automated task (i.e., name the ink colors) than the more automated task of reading the words. The number of correct responses in the 45 seconds taken to complete 10 x 10 columns in each card was noted. Different components of cognition like attention, speed, cognitive flexibility, and working memory can be assessed using this test [5].

Trail-Making Test (TMT)

TMT consists of two parts; Part A requires the participant to draw lines on the page connecting 25 encircled numbers sequentially, which are randomly distributed on the sheet as quickly as possible. Similarly, in Part B, participants are asked to connect alphabets and numbers alternatively (e.g., 1, A, 2, B, 3, C). Performance is assessed based on the time taken to complete the trail made by the numbers on the test in ascending order without lifting the pen/pencil. If the subject makes an error, it can be pointed out and corrected. The time taken to correct the error is added to the completion time. TMT Part B is a better indicator of working memory, executive control, and cognitive flexibility [6].

Statistical analysis

Statistical analysis was performed by using SPSS Statistics version 16 (IBM Corp., Armonk, NY). The Kolmogorov-Smirnov test was used to test the normal distribution. All the values were presented as mean ± SD. The unpaired student's t-test was used to examine the gender difference in the adoption of SRL strategies and cognitive flexibility. The Pearson correlation was used to correlate SRL strategies and cognitive flexibility. A p-value of less than 0.05 was considered statistically significant.

## Results

Demographic details of the participants are presented in Table 1. Among the 220 participants, the mean age and BMI were 21.76 ± 1.77 years, and 21.06 ± 1.25 kg/m^2^ respectively, indicating that all participants belonged to the same age group (young adults) and had a normal BMI. As shown in Table 2, there was no significant difference in terms of gender in any of the subscale scores of learning strategy: rehearsal (p=0.838), elaboration (p=0.929), organization (p=0.819), critical thinking (p=0.669), metacognition (p=0.902), time and study environment (p=0.247), effort regulation (p=0.676), peer learning (p=0.903), and help-seeking (p=0.856). In cognitive flexibility tests, the number of correct responses obtained for Card W (p=0.807), Card C (p=0.596), and Card CW (p=0.792) of SCWT within 45 seconds also did not show any significant difference between male and female students. There was no significant difference with regard to gender in TMT Part A (p=0.461) and Part B (p=0.135) either.

**Table 1:** Mean age and BMI of the participants BMI: body mass Index; SD: standard deviation

Demography	Mean	SD
Age (years)	21.76	1.77
BMI (kg/m^2^)	21.06	1.25

**Table 2:** Genderwise difference in learning strategies and cognitive flexibility tests SD: standard deviation

Variable	Male, mean ± SD	Female, mean ± SD	P-value
Cognitive and metacognitive strategies			
Rehearsal	4.562 ± 1.0816	4.490 ± 1.3702	0.838
Elaboration	4.254 ± 0.8470	4.281 ± 1.2050	0.929
Organization	4.698 ± 1.1131	4.779 ± 1.3552	0.819
Critical thinking	4.433 ± 1.1138	4.585 ± 1.3505	0.669
Metacognition	4.146 ± 0.9371	4.181 ± 1.0415	0.902
Resource management strategies			
Time and study environment	4.075 ± 1.1280	4.435 ± 1.0396	0.247
Effort regulation	4.219 ± 1.0893	4.087 ± 1.1335	0.676
Peer learning	4.229 ± 1.1667	4.269 ± 1.1547	0.903
Help-seeking	4.260 ± 1.1265	4.183 ± 1.7868	0.856
Stroop Color and Word Test			
Card W (number responded)	98.6667 ± 3.37080	98.8846 ± 2.88897	0.807
Card C (number responded)	82.0000 ± 13.78089	79.9615 ± 13.24381	0.596
Card CW (number responded)	73.3750 ± 14.95446	72.2308 ± 15.45136	0.792
Trail Making Test			
Trail A (time in seconds)	23.7083 ± 6.74685	25.0769 ± 6.28600	0.461
Trail B (time in seconds)	44.0000 ± 13.84699	38.3462 ± 12.46095	0.135

Table 3 summarizes the mean subscale scores of all the participants in cognitive and metacognitive strategies, as follows - rehearsal: 4.53 ± 1.29, elaboration: 4.27 ± 1.04, organization: 4.74 ± 1.23, critical thinking: 4.51 ± 1.23, and metacognition: 4.16 ± 0.98. The mean subscale scores of all the participants in resource management strategies were as follows: 4.26 ± 1.09 for time and study environment, 4.15 ± 1.10 for effort regulation, 4.25 ± 1.15 for peer learning, and 4.22 ± 1.49 for help-seeking. Hence, the mean score of the participants remained above 3 in all the learning strategy items. As shown in Table 4, the mean scores in the Stroop test for Card W, Card C, and Card CW were as follows: 98.78 ± 3.10, 80.94 ± 13.40, and 72.78 ± 15.07 respectively. In TMT, the mean latency (in seconds) for Trail A was 24.42 ± 6.48 and that for Trail B was 41.06 ± 13.32. These results show that as the complexity of the task increases, the number of correct responses decreases and the latency to respond increases.

**Table 3:** Mean scores for learning strategy adopted by students of both gender The number within brackets in the first column indicates the number of items assessed in each subscale SD: standard deviation

Subscale	Mean	SD
Cognitive and metacognitive strategies		
Rehearsal (4)	4.53	1.29
Elaboration (6)	4.27	1.04
Organization (4)	4.74	1.23
Critical thinking (5)	4.51	1.23
Metacognition (12)	4.16	0.98
Resource management strategies		
Time and study environment (8)	4.26	1.09
Effort regulation (4)	4.15	1.10
Peer learning (3)	4.25	1.15
Help-seeking (4)	4.22	1.49

**Table 4:** Mean scores for Stroop Color and Word Test and Trail-Making Test (cognitive flexibility tests) among students of both genders SD: standard deviation

Cognitive flexibility tests	Mean	SD
Card W (number responded)	98.78	3.10
Card C (number responded)	80.94	13.40
Card CW (number responded)	72.78	15.07
Trail A (time in seconds)	24.42	6.48
Trail B (time in seconds)	41.06	13.32

Table 5 demonstrates the Pearson correlation analysis between cognitive flexibility tests and learning strategy scores. Responses to the Card W test did not show a significant correlation with any of the learning strategy scores since it is a very basic test. However, responses to Card C and Card CW of the Stroop test showed a significant positive correlation (p<0.001) with subscale scores of learning strategies. In TMT, the latency of Trail A showed a significant negative correlation (p<0.001) with all the subscale scores of the learning strategies, and the latency of Trail B showed a negative correlation with rehearsal (p=0.03), organization (p=0.03), and effort regulation strategies (p=0.01) of MSLQ.

**Table 5:** Correlation between cognitive flexibility tests and learning strategies among students *P<0.05. **P<0.001 MSLQ: Motivated Strategy for Learning Questionnaire

MSLQ and cognitive flexibility		Stroop test - Card W	Stroop test - Card C	Stroop test - Card CW	Trail Making Test - Part A	Trail Making Test - Part B
Rehearsal	r	-0.088	0.584**	0.665**	-0.564**	-0.294*
	P	0.542	0.000	0.000	0.000	0.03
Elaboration	r	-0.098	0.606**	0.651**	-0.475**	-0.136
	P	0.498	0.000	0.000	0.000	0.34
Organization	r	0.029	0.612**	0.630**	-0.543**	-0.294*
	P	0.843	0.000	0.000	0.000	0.03
Critical thinking	r	-0.077	0.303*	0.379**	-0.318*	-0.138
	P	0.596	0.033	0.007	0.025	0.33
Metacognition	r	-0.051	0.693**	0.790**	-0.690**	-0.242
	P	0.726	0.000	0.000	0.000	0.09
Time and study environment	r	-0.049	0.478**	0.606**	-0.567**	-0.162
	P	0.738	0.000	0.000	0.000	0.26
Effort regulation	r	-0.008	0.634**	0.685**	-0.654**	-0.332*
	P	0.956	0.000	0.000	0.000	0.01
Peer learning	r	-0.185	0.344*	0.500**	-0.443**	-0.208
	P	0.199	0.015	0.000	0.001	0.14
Help-seeking	r	-0.122	0.500**	0.633**	-0.629**	-0.103
	P	0.400	0.000	0.000	0.000	0.47

## Discussion

There was no significant difference in the subscale scores of MSLQ and cognitive flexibility among male and female medical students. The mean scores of all the students were found to be more than 4 in all nine subscales of MSLQ. The mean number of responses decreased as the students performed Card W, Card C, and Card CW of SCWT, and the time taken increased in TMT Part B compared to Part A. There was a positive significant correlation between SCWT (Card C and Card CW) and SRL strategy and a negative correlation between TMT and SRL. Similar to our study, Nikoopour et al. reported that gender has no impact on self-regulation [7]. In contrast, a study by Liu et al. among high school students reported that females were found to be better at SRL compared to males [8]. Another study reported higher levels of cognitive flexibility among males compared to females [9]. Thus, insignificant gender differences in our study might be attributed to common eligibility criteria adopted for MBBS admission through NEET (National Eligibility cum Entrance Test).

Each subscale on MSLQ assesses the strength of the subject’s learning ability [4]. In terms of cognitive and metacognitive strategy, the subscale rehearsal pertains to working memory, attention, and encoding of information. The subscale elaboration informs the learner's long-term memory and their ability to connect and correlate information. The subscale organization throws light on students’ ability to participate in active learning. The critical thinking subscale provides knowledge regarding the decision-making and problem-solving abilities of the students. Students' ability to plan, monitor, and regulate their cognition can be assessed by the metacognition subscale.

With regard to resource management strategy, the time and study environment subscale analyzes the student's ability to use study time effectively and make their environment study-friendly. Effort regulation provides information on students' commitment to completing their tasks without distractions. Peer learning looks into students' attitudes toward helping each other with regard to academic achievement. The ability of the students to approach their seniors, peers, and mentors for learning management is analyzed using the help-seeking subscale.

Our results showed that students who adopt SRL strategies score better on SCWT, which indicates better cognitive flexibility. Previous studies have also reported that SRL strategies improved academic performance [10] and led to higher levels of self-satisfaction and awareness [10]. Rui et al. emphasized that the development of metacognitive strategy enhanced active learning among students [11]. In line with our results, Chitra et al. found that the practice of SRL strategies enabled students to be more successful in their careers [12]. Siddiqui et al. have reported that SRL strategies not only enhance medical students' performance as care providers and lifelong learners but also equip them to manage difficult situations by enhancing their leadership qualities, communication, and decision-making skills [13].

Neuroimaging studies have reported that cognitive flexibility involves the repeated firing of the lateral frontoparietal network and the mid-cingular-insular network [14,15]. SRL strategies may enhance brain cell activity and synaptic plasticity via a neurofeedback training mechanism [16]. The study findings show that self-regulation is negatively correlated with the time taken in TMT. Hence, enhanced self-regulation improves the swiftness and accuracy in trail making. TMT Part B was found to be more sensitive in assessing cognitive flexibility. In our results, the subscale rehearsal, organization, and effort regulation were significantly associated with TMT Part B. Hence, students can be particularly encouraged to practice these three strategies to enhance their cognitive flexibility. However, Rhodes et al. have reported that elaboration strategy enhanced cognitive flexibility among undergraduate students [17]. Jiatong et al. have reported that promoting cognitive flexibility among medical students encourages them to pursue an entrepreneurial career that could help build the Indian economy and promote the society's health and wealth [18]. As per Ballouk et al., revising the design of the medical curriculum by incorporating SRL can augment students’ academic capabilities and clinical skills [19].

In summary, it was found that medical students are better at adopting SRL strategies, and these strategies enhance their cognitive flexibility [20]. They can be further guided by their mentors on SRL strategies that positively correlate with cognitive flexibility to deal with future emergency clinical situations or pandemics like COVID-19 that have affected the entire educational system. 

This study has a few limitations. The influence of students’ learning environment (hostelite or day-scholar) and socioeconomic status was not analyzed. Also, we believe the study could have provided more insightful findings if we had compared advanced and slow learners in terms of the cognitive flexibility and learning strategies practiced. Hence, we recommend further multicentric studies to enhance the generalizability of our findings. Researchers should also consider performing interventional studies where specific SRL strategies are applied to determine the improvement in cognitive flexibility.

## Conclusions

Based on our findings, the ultimate benefit of implementing SRL methods is that it helps students save a lot of valuable time. This would enable them to make better use of their time. Hence, initiatives to promote and enhance SRL can be implemented among students in need as these can augment not only their academic performance but also their cognitive flexibility.
